# Coagulopathy Induced by Antibiotics Usage and Bowel Obstruction With Colon Cancer: Report of a Rare Case

**DOI:** 10.7759/cureus.52865

**Published:** 2024-01-24

**Authors:** Sho Fujiwara, Ryujiro Akaishi, Tomoki Yokosawa, Hiroshi Suzuki, Toru Hoshida

**Affiliations:** 1 Surgery, Iwate Prefectural Ofunato Hospital, Ofunato, JPN; 2 Surgery, Columbia University Irving Medical Center, New York, USA; 3 Emergency and Critical Care, Iwate Prefectural Ofunato Hospital, Ofunato, JPN

**Keywords:** postoperative ileus, vitamin k deficiency, prolonged fasting, antibiotics awareness, coagulopathy

## Abstract

This case report presents a rare occurrence of coagulopathy induced by antibiotics in a woman in her 90s with chronic bowel obstruction and massive colon cancer. The patient developed vitamin K deficiency-related coagulopathy following antibiotic administration, resulting in bleeding complications. Despite initial consideration of disseminated intravascular coagulation, further investigations revealed antibiotic-induced vitamin K deficiency. Prompt discontinuation of antibiotics and IV vitamin K2 administration led to the resolution of coagulopathy. The case emphasizes the importance of cautious antibiotic use in patients with chronic bowel obstruction and prolonged fasting. The protein induced by vitamin K absence-II (PIVKA-II) proved valuable in diagnosing vitamin K deficiency. The learning points include the potential for coagulopathy with antibiotics in prolonged bowel obstruction and the utility of PIVKA-II in assessing vitamin K deficiency. Healthcare providers should exercise caution when administering antibiotics in similar clinical scenarios.

## Introduction

Vitamin K deficiency can lead to coagulopathy in some clinical scenarios. Some major causes of vitamin K deficiency are decreased intake or absorption of vitamin K, altered biosynthesis in intestinal bacteria, and inhibition of liver metabolism [1-3]. Antibiotics with N-methylthiotetrazole (NMTT) side chain could risk vitamin K deficiency by inhibiting vitamin K epoxide reductase like warfarin. In addition, chronic intestinal blockage and fasting for more than seven days can also reduce vitamin K absorption [1]. However, vitamin K deficiency and coagulopathy in adults are rare because adults have enough storage and intake of vitamin K, although it is common in neonates [4]. Here, we present a case of coagulopathy with vitamin K deficiency caused by these multiple risk factors: chronic bowel obstruction due to colon cancer and preoperative use of antibiotics with NMTT side chain. 

## Case presentation

**Figure 1:**
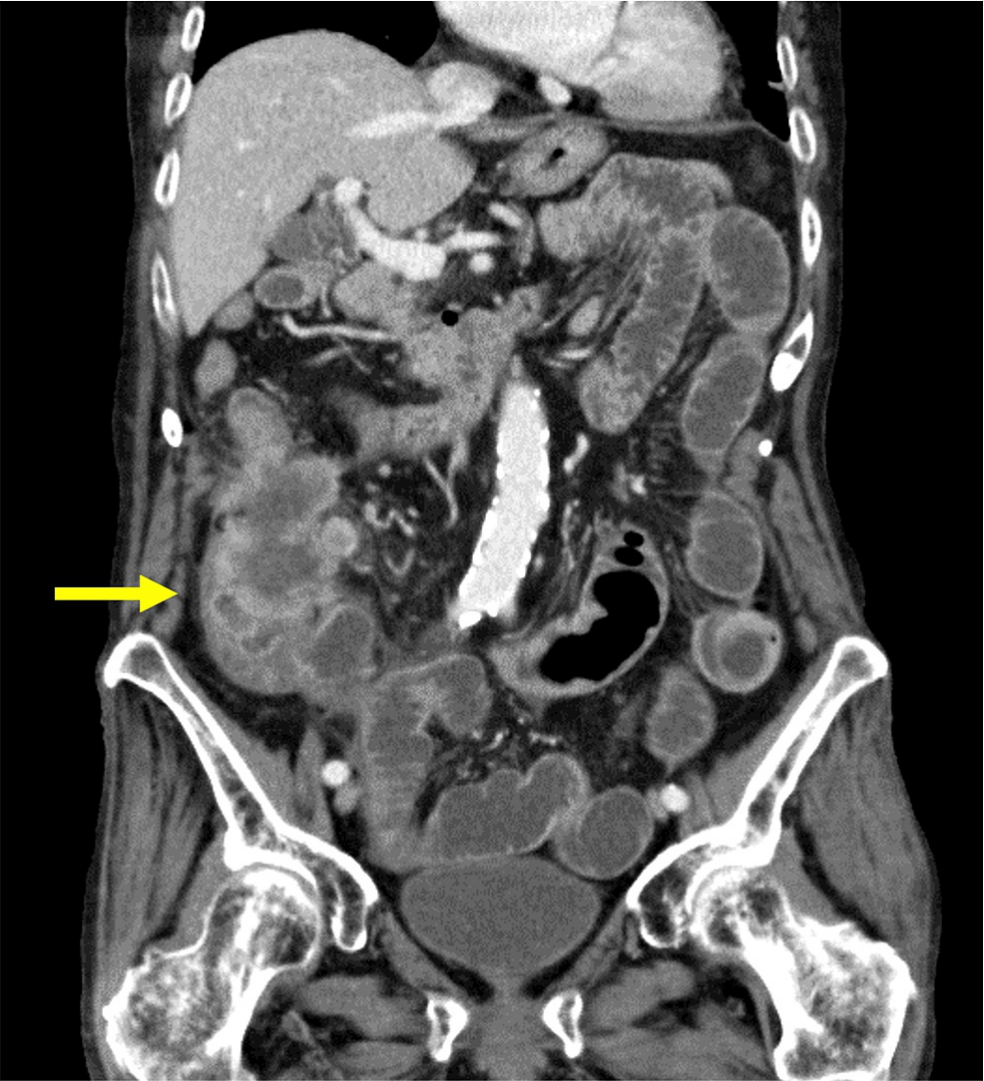
Preoperative abdominal contrast-enhanced CT Abdominal contrast-enhanced CT revealed massive colon cancer with invasion of the terminal ileum (yellow arrow) and bowel obstruction.

A female patient in her nineties with a medical history of hypertension presented with vomiting and distension. The symptoms began three days ago, and the family physician prescribed 5 mg of metoclopramide and 330 mg of magnesium oxide orally. However, she was not able to eat well seven days ago. The patient regularly takes 20 mg of esomeprazole for esophageal reflux and 40 mg of telmisartan for hypertension. A physical examination revealed abdominal distension and a palpable tumor in the right lower quadrant. Abdominal contrast-enhanced CT revealed colon cancer invading the terminal ileum and causing bowel obstruction (Figure 1).

**Figure 2:**
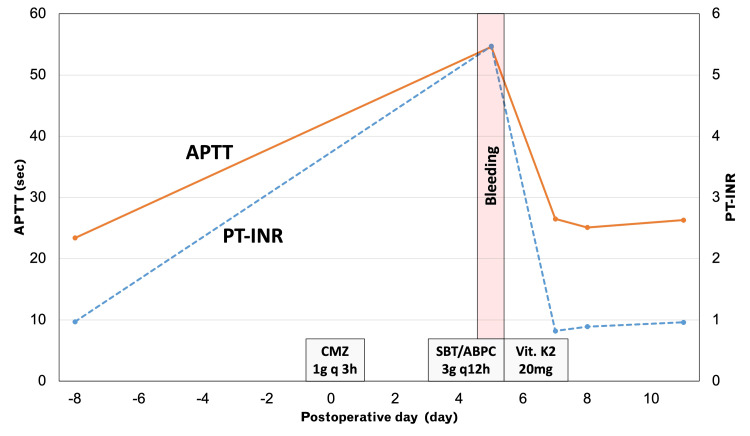
Time courses of the coagulation tests On postoperative day five, the patient received 3 g of ampicillin/sulbactam intravenously every 12 hours due to aspiration pneumonia. After receiving three rounds of antibiotics, the patient developed melena and bleeding from a surgical wound. APTT: activated partial thromboplastin time; PT-INR: prothrombin time and international normalized ratio

To palliatively relieve the obstruction, we performed ileocolic side-to-side anastomosis, considering the patient's performance status. During the surgery, we administered prophylactic antibiotic cefmetazole (1 g) intravenously every three hours. Although the patient had a bowel movement on postoperative day (POD) three, she experienced vomiting due to ileus and developed aspiration pneumonia on POD four. Subsequently, the patient was administered 3 g of ampicillin/sulbactam intravenously every 12 hours. On POD five, the patient experienced melena and bleeding from a surgical wound after three administrations of antibiotics. A blood test revealed a prothrombin time and international normalized ratio (PT-INR) of 5.47 and an activated partial thromboplastin time (APTT) of 54.6 seconds (Figure 2). 

Initially, we considered disseminated intravascular coagulation (DIC) as the cause of coagulopathy due to bacterial translocation caused by chronic dilated bowel obstruction. However, the platelet count, fibrinogen level, and fibrin/fibrinogen degradation products were within the normal range. Subsequently, we evaluated vitamin K deficiency by examining the protein induced by vitamin K absence-II (PIVKA-II). The patient was diagnosed with antibiotic-induced vitamin K deficiency as PIVKA-II was 33,000 mAU/mL. Consequently, we discontinued the antibiotics and administered IV vitamin K2 20 mg for two days. The coagulopathy improved on POD seven. INR and APTT were within normal range without additional treatment for coagulopathy. Oral taking started on POD 10 and the patient was discharged from our hospital on POD 18 without requiring a blood transfusion.

## Discussion

Antibiotic-induced vitamin K deficiency is a rare cause of coagulopathy. The normal reference range of vitamin K is 0.8-5.4 ng/mL and the dietary requirement of vitamin K is 1 μg/kg/day [5,6]. There are several major causes of vitamin K deficiency, including decreased intake or absorption of vitamin K, alteration of vitamin K biosynthesis in gut bacteria, and inhibition of the metabolic cycle of vitamin K in the liver [1]. Metabolic inhibition is induced by cephalosporins with an NMTT side-chain, inhibiting vitamin K epoxide reductase, similar to warfarin. Antibiotics that contain the NMTT or 2-methyl-1,3,4-thiadiazole side chain, such as cefamandole, cefmenoxime, cefoperazone, cefotetan, moxalactam, and cefazolin, inhibit the γ-carboxylation and activation of vitamin K-dependent clotting factors. They also decrease liver microsomal vitamin K-2,3-epoxide activity. These inhibitions can induce coagulopathy in vitamin K deficiency [7].

In this case, ampicillin/sulbactam was considered to be safer than cephalosporins because it does not contain the NMTT side chain [8], but caution should still be exercised due to the lack of oral intake for more than seven days and chronic intestinal obstruction leading to decreased vitamin K absorption [1]. Moreover, the administration of ampicillin/sulbactam can alter the intestinal bacteria. It is important to note that cefmetazole, which was administered as a prophylactic antibiotic, contains an NMTT side chain that could exacerbate coagulopathy [8]. These factors can lead to vitamin K deficiency and coagulopathy. Therefore, administering antibiotics to patients with chronic bowel obstruction, advanced colorectal cancer, and long-term absence of oral intake requires caution. PIVKA-II is a useful tool for evaluating vitamin K deficiencies [9]. If DIC is ruled out and fresh frozen plasma is considered, vitamin K2 should be administered intravenously [10]. There are no reports on whether vitamin K1 or vitamin K2 is appropriate for this case. However, we used vitamin K2 intravenously because vitamin K2 is absorbed from the intestine and produced by intestinal flora. In this case, bowel obstruction and changing microbiota could worsen the vitamin K2 deficiency [11]. Thus, vitamin K2 was supposed to be more appropriate.

## Conclusions

Even if we use ampicillin/sulbactam, which does not contain the NMTT side chain, we still need to be careful about vitamin K deficiency if we use antibiotics with the NMTT side chain as a prophylactic for surgery after a long period of bowel obstruction and fasting. To evaluate a deficiency in vitamin K, PIVKA-II can be used. After ruling out DIC and other causes of coagulopathy, the administration of Vitamin K2 should be considered.
